# Clinical Efficacy and Meta-Analysis of Stem Cell Therapies for Patients with Brain Ischemia

**DOI:** 10.1155/2016/6129579

**Published:** 2016-08-31

**Authors:** Lukui Chen, Guilong Zhang, Ahsan Ali Khan, Xiaoyuan Guo, Yuchun Gu

**Affiliations:** ^1^Department of Neurosurgery, Zhongda Hospital, School of Medicine, Southeast University, Nanjing 210009, China; ^2^Aston Institute of Regenerative Medicine, Aston Medical School, Aston University, Birmingham, UK

## Abstract

*Objective*. Systematic review and meta-analysis to observe the efficacy and safety of stem cell transplantation therapy in patients with brain ischemia.* Methods*. We searched Cochrane Library, PubMed, Ovid, CBM, CNKI, WanFang, and VIP Data from its inception to December 2015, to collect randomized controlled trials (RCT) of stem cell transplantation for the ischemic stroke. Two authors independently screened the literature according to the inclusion and exclusion criteria, extracted data, and assessed the risk of bias. Thereafter, meta-analysis was performed.* Results*. Sixteen studies and eighteen independent treatments were included in the current meta-analysis. The results based upon the pooled mean difference from baseline to follow-up points showed that the stem cell transplantation group was superior to the control group with statistical significance in the neurologic deficits score (NIHSS, MD = 1.57; 95% CI, 0.64–2.51; *I* ^2^ = 57%; *p* = 0.001), motor function (FMA, MD = 4.23; 95% CI, 3.08–5.38; *I* ^2^ = 0%; *p* < 0.00001), daily life ability (Barthel, MD = 8.37; 95% CI, 4.83–11.91; *I* ^2^ = 63%; *p* < 0.00001), and functional independence (FIM, MD = 8.89; 95% CI, 4.70–13.08; *I* ^2^ = 79%; *p* < 0.0001).* Conclusions*. It is suggested that the stem cell transplantation therapy for patients with brain ischemic stroke can significantly improve the neurological deficits and daily life quality, with no serious adverse events. However, higher quality and larger data studies are required for further investigation to support clinical application of stem cell transplantation.

## 1. Introduction

Ischemic stroke, also known as cerebrovascular accident, is caused by the decreased or interrupted blood supply in the part of the brain. It is of the dominant diseases affecting human health and causing the most mortalities, together with coronary heart disease and cancer [1]. There are more than fifty million people suffering with varying degrees of ischemic stroke in the world; the rate of deaths was close to 10% each year; meanwhile most survivors were disabled, and not only did it affect patients physically, but also there are huge impacts economically and to spirit of patients and their families [1, 2]. The main pathological manifestation of ischemic stroke is the ischemic brain tissue (hypoxia/necrosis) for a short time, and it results in reduced number of neurons, interrupted neural axon network, and formation of local free oxygen radical species, leading to harsh environments in the peripheries of ischemic region, damaging brain self-healing, and eventually resulting in the permanent loss of nerve tissue or disabling of brain function. Currently there is no efficient therapy after stroke, except tPA (tissue plasminogen activator) which is the only efficient treatment drug in clinical settings [3]. However, it plays a major role in the early stage of ischemia, which has a very short time window (4.5 hours, and less than 6 hours) and can increase the risk of cerebral hemorrhage. In addition, only approximately 5% of stroke patients can receive this treatment in the United States [4].

In recent decades, with the rapid development of stem cell research, many types of stem cells (including adult stem cells) give rise to more and more attentions in clinic and showed that transplanted stem cells could promote therapies for ischemic stroke [5, 6]. Stem cells have high self-replication and self-differentiation potentials and can differentiate into many types of cells, such as neural stem cells (NSC) which may further differentiate into neurons, astrocytes, and oligodendrocytes, and so forth [7], with lower immunogenicity and better histocompatibility. It has been considered as good tools and the most promising natural resources for the treatment of brain stroke [8]. Although many studies regarding stem cells transplantation therapies have been carried out in experimental models or preclinically, because of safety, ethical, and therapeutic effect issues, there is still a long distance for clinical application of stem cells.

Therefore, the purpose of the current study is to systematically review and evaluate the potential efficacy and safety of stem cell transplantation therapy for patients with ischemic brain stroke. We collected the randomized controlled trials (RCT) of stem cell transplantation for ischemic stroke in recent 20 years and used systematical review and meta-analysis approaches to identify the possible publication bias and to investigate the impact of various aspects of reporting outcomes in clinical ischemic stroke studies.

## 2. Methods

### 2.1. Search Strategy

We searched the literatures in Cochrane Library, PubMed, Ovid, CBM, CNKI, WanFang, and VIP Data from its inception to December 2015, to collect randomized controlled trials (RCT) of stem cell transplantation for ischemic stroke. Published languages contain English and Chinese. The search words contain stem cell, transplantation, brain ischemia, and cerebrovascular stroke, and the details of search strategy are described in the following list (take PubMed, for instance):

 #1 brain ischemia OR cerebrovascular stroke OR ischemic stroke.
 #2 stem cell OR stem cell transplantation.
 #3 transplantation.
 #4 #1 AND #2 AND #3 (filters: randomized controlled trial; limited: humans).

### 2.2. Inclusion and Exclusion Criteria

Intervention measurements contain all types of stem cells, such as neural stem cell (NSC), bone marrow mesenchymal stem cell (MSC), and umbilical cord mesenchymal stem cell (UC-MSC). Research subjects (ischemic stroke patients) must meet WHO diagnostic criteria of brain stroke and exclude cerebral hemorrhage by brain CT or MRI. Each group must have more than 5 patients. The outcomes of treatment from baseline to follow-up points (or before/after treatment) should contain National Institutes of Health Stroke Scale (NIHSS), Fugl-Meyer Assessment (FMA), Barthel index, or Functional Independence Measure (FIM). We also excluded literature reviews, meta-analysis, meeting abstracts, case reports, repeated studies, experimental model researches, and other diseases researches.

### 2.3. Data Extraction

Two authors independently screened the literatures according to the inclusion and exclusion criteria, extracted data, and assessed the risk of bias. We extracted these data including authors, year of publication, patients' age, gender, time/type of cerebral ischemia, number of patients (treatment group/control group), type of SCs (stem cells) intervention, intervention dose, time of administration, route of delivery, and outcome assessments, mean, SD (standard deviation) or SE (standard error), and adverse effects. We defined the treatment comparison as the outcomes in the control group compared to the treated group. If more than one intervention was given in one study, we regarded these to be another independent intervention. Furthermore, if the treatment was administered in multiple doses, we considered the sum of all doses administered. Finally, if the functional outcomes were reported for >1 time points, we only included the last time of assessment. We used the manual of Cochrane systematic review (5.1.0 RCT bias risk assessment tools) to evaluate the bias risk of all included studies.

### 2.4. Data Analysis/Statistical Analysis

The pooled outcome difference of stem cell transplantation in the treatment of ischemic stroke between each treatment group and the control group used the quantitative data of mean difference (MD) or standardized mean difference (SMD) and 95% confidence interval (CI) to meta-analysis (RevMan 5.3 software). Heterogeneity between the results of included studies was analyzed using *χ* ^2^ test (significance level *α* = 0.05) and quantitatively determined the size of heterogeneity by combining with *I* ^2^. If no significant heterogeneity or little heterogeneity between studies was found, the fixed effects model was then used. If the large statistical heterogeneity between studies was observed, the random effects model was then used after excluding the significant clinical heterogeneity. When the heterogeneity was obvious, the subgroup analysis or sensitivity analysis was used, or just descriptive analysis. Finally, we used the funnel plot analysis to evaluate the publication bias.

## 3. Results

### 3.1. Study Characteristics

**Figure 1:**
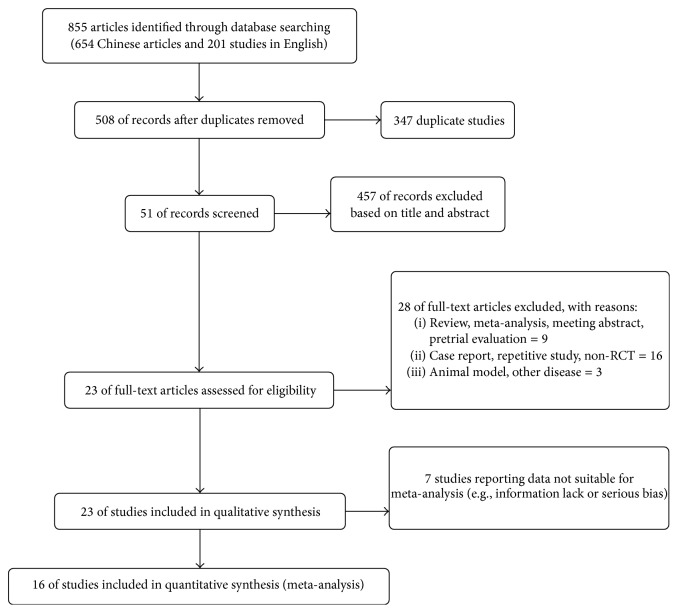
Flow diagram showing summary of study selection procedure.

We identified a total of 23 studies from 855 publications (654 Chinese articles and 201 studies in English) for this systematic review. The number of patients is 680 in the stem cell treatment group and 694 in the control group, respectively. However, there are seven researches which were excluded due to lack of information or serious bias, and finally 16 studies (18 independent interventions) were conducted in the meta-analysis (Figure 1).

There were four interventions with NSC transplantation, one with UC-MSC, one with PBSC, and twelve combined with MSC transplantation. There were 6 out of 18 interventions which were performed in acute cerebral ischemia, 7 were done in chronic cerebral ischemic states, and time of treatment was not mentioned in remaining 5 interventions. Furthermore, there were 5 of 18 treatments which were injected in subarachnoid space, 1 through carotid artery, 1 by intracerebral transplantation, and 11 interventions administrated intravenously. The duration of follow-up varied from 1 month to 12 months (Table 1).

### 3.2. Quality Assessment and Bias Risk

Most studies had a certain risk of bias, especially the Chinese articles in which the trial design was not rigorous and had more defects in the blinding and data's integrity. The English studies have less bias risk than Chinese articles due to the trial design which followed the RCT policy (Table 2).

### 3.3. Meta-Analysis and Effect Evaluation

**Figure 2:**
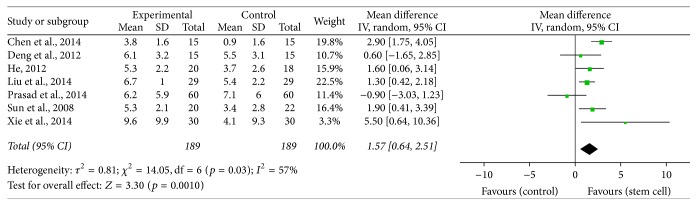
The effect size of NIHSS improvement across studies by meta-analysis.

**Figure 3:**
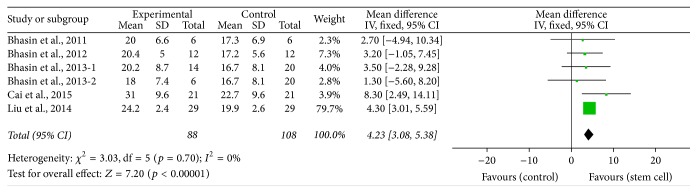
The effect size of improved FMA across studies by meta-analysis.

**Figure 4:**
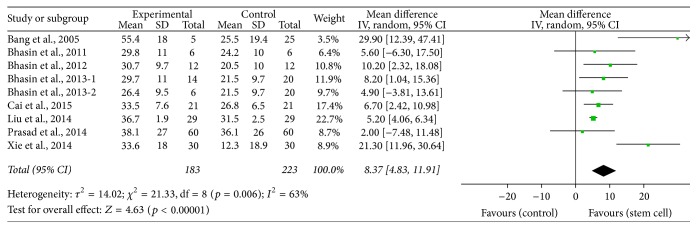
The effect size of Barthel index improvement across studies by meta-analysis.

**Figure 5:**
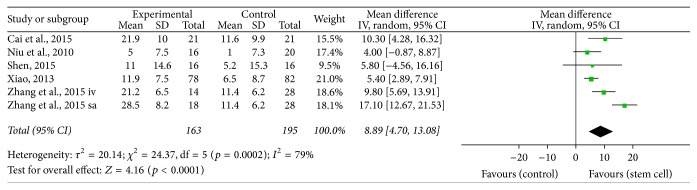
The effect size of improved FIM across studies by meta-analysis.

We performed the pooled data for meta-analysis and found that 7 interventions reported NIHSS improvement [10, 13, 14, 18–21], 6 interventions determined FMA functional optimization [12, 15, 17, 18, 24], 9 treatments pointed out Barthel index enhancement [9, 12, 15, 17–19, 21, 24], and 6 treatment groups showed FIM functional improvement [11, 16, 22–24]. The results of meta-analysis were as follows: NIHSS (MD = 1.57, 95% CI 0.64–2.51, *I* ^2^ = 57%, *p* = 0.001, Figure 2), FMA (MD = 4.23, 95% CI 3.08–5.38, *I* ^2^ = 0%, *p* < 0.00001, Figure 3), Barthel (MD = 8.37, 95% CI 4.83–11.91, *I* ^2^ = 63%, *p* < 0.00001, Figure 4), and FIM (MD = 8.89, 95% CI 4.70–13.08, *I* ^2^ = 79%, *p* < 0.0001, Figure 5). All the outcomes after the merger were in favor of stem cell transplantation group, and each of the differences was statistically significant. Only four studies reported adverse effects, which included mild fever or headache and these were self-relieved within a short period.

Then we performed subgroup analysis for further evaluation of the clinical efficacy based on several clinical variables, and we focused on four clinical variables: time/type of cerebral ischemia, type of SCs interventions, route of delivery, and follow-up period. Each clinical variable was analyzed by interacting with NIHSS or Barthel index which were reported consistently, including evaluation of subgroup effects and heterogeneity analysis (Tables 3 and 4), and the results were in favor of the nonvein injected group (e.g., subarachnoid space and carotid artery administration) and long-time follow-up period (more than 6 months). However, when the time and type of cerebral ischemia were analyzed, NIHSS and Barthel index were improved in chronic cerebral ischemia and acute cerebral ischemic stroke patients, respectively. Due to the reason that most studies used MSC transplantation, the effect of SCs type interventions could not be obtained.

## 4. Discussion

Stem cells have several special biological characteristics, such as proliferation ability, multidirectional differentiation, and good histocompatibility, and numerous studies have focused on the therapeutic potential of stem cell therapy for various refractory diseases. With the rise of stem cell therapy in recent years, different types of stem cells have been widely investigated and applied for ischemic stroke therapy, especially with a sharp increase in NSC and MSC transplantation. Lees et al. evaluated 117 studies on preclinical experimental model of stem cell therapy by meta-analysis and showed that stem cell transplantation had improved the animals' neural function and reduced the area of cerebral infarct [25]. Vu et al. studied the efficacy of preclinical mesenchymal stromal cells transplantation therapy for ischemic stroke models and demonstrated the favorable (MSC) outcomes, and the outcomes were related with the type of MSC source, route of delivery, time of injection, and intervention dose [26]. Because of safety, efficacy, and ethical reasons, there still remains a long way before the stem cell therapy can be actually used in clinical settings. Liu et al. searched the MSC transplantation therapy for cerebral ischemic patients and found 6 studies (332 patients); they confirmed that MSC transplantation could significantly improve patients' neural functional defects, motor functions, and daily life abilities [27]. Jeong et al. analyzed the safety and efficacy of stem cell transplantation therapy for brain stroke; they included 14 researches which independently use stem cell therapy (not RCT studies) and determined that stem cell therapy improved the patients' grades of NIHSS, Barthel index, and Rankin functions [28]. All those studies confirmed the therapeutic potential of stem cells clinically.

Here we have searched the RCT studies of stem cell transplantation for ischemic stroke and used meta-analysis approach to identify the possible publication bias and to investigate the clinical efficacy of reported outcomes (NIHSS, FMA, Barthel and FIM) in ischemic stroke studies. A total of 23 studies recruited 1374 patients, and the patients of ischemic stroke must have met WHO diagnostic criteria of brain stroke. Cerebral hemorrhage was ruled out in these patients by brain CT or MRI. Each group must have more than 5 patients. The type of interventions contained NSC, MSC, UC-MSC, and PBSC. Finally, 16 articles and 18 treatment interventions have been analyzed by meta-analysis; the outcomes of treatment from baseline to follow-up points included NIHSS, FMA, Barthel index, or FIM which all improved to varying degrees, with fewer adverse effects. Then we used 4 clinical variables (time/type of cerebral ischemia, type of SCs interventions, route of delivery, and follow-up period) to interact with 2 outcome indicators (NIHSS and Barthel) and found that patients who received SCs transplantation via subarachnoid space, carotid artery, and intracerebral were superior to those in which SCs were administered intravenously, and long-term follow-up was associated with better outcome than short-term follow-up. But there was no relation with time and type of cerebral infarct or type of SC interventions with functional improvement. Due to the fact that most studies had defects in blinding, data's integrity, and trial designs, our meta-analysis might have some risks of bias.

In conclusion, the current meta-analysis of clinical studies of SCs therapy for ischemic stroke demonstrated significant and favorable effects on behavioral, motor, and life quality outcomes. However, because of few clinical trials for stem cell transplantation therapy, lower frequency of patient participation, and possible bias issues in trial designs, there is possibility of bias occurrence. Therefore, further high quality and bigger data studies are needed in the future to investigate the possibilities of stem cells transplantation in clinical settings.

## Figures and Tables

**Table 1:** Study characteristic report.

Author	Year of publish	Numbers (SCs/Con)	Age	Type of stroke Acute/chronic	Gender Male/female	Type of interventions		Cells dose ×10^6^	Route of injection	Follow-up (month)	Outcome effect indicators
						Stem cell	Control				
Bang et al. [9]	2005	5/25	63.0 ± 7.5	Acute	18/12	MSC	Routine	100	Intravenous	12	Barthel
Sun et al. [10]	2008	20/22	57.8 ± 8.9	X	32/10	MSC	Routine	X	Intravenous	3	NIHSS
Niu et al. [11]	2010	16/20	56 ± 7	Chronic	22/14	NSC	Routine	X	Subarachnoid	6	FIM
Bhasin et al. [12]	2011	6/6	42	Chronic	8/4	MSC	Routine	50–60	Intravenous	6	FMA, Barthel
Deng et al. [13]	2012	15/15	X	Acute	X	MSC	Routine	10–50	Intravenous	1	NIHSS
He [14]	2012	20/18	56.4 ± 7.9	X	23/15	MSC	Routine	100	Intravenous	3	NIHSS
Bhasin et al. [15]	2012	12/12	46.5	Chronic	X	MNC	Routine	50–60	Intravenous	6	FMA, Barthel
Xiao [16]	2013	78/82	56.9 ± 9.2	X	103/57	NSC	Routine	8000	Subarachnoid	X	FIM
Bhasin et al. [17]	2013	14/20	45.1 ± 12.1	Chronic	32/8	MNC	Routine	50–60	Intravenous	6	FMA, Barthel
		6/20	45.1 ± 12.1	Chronic	32/8	MSC	Routine	50–60	Intravenous	6	FMA, Barthel
Liu et al. [18]	2014	29/29	55.3 ± 3.6	Acute	38/20	MSC	Routine	100	Subarachnoid	3	NIHSS, Barthel, FMA
Xie et al. [19]	2014	30/30	51.4 ± 7.2	Acute	37/23	MSC	Routine	X	Subarachnoid	6	NIHSS, Barthel
Chen et al. [20]	2014	15/15	50.1 ± 7.7	Chronic	20/10	PBSC	Routine	3–8	Stereotactic	12	NIHSS
Prasad et al. [21]	2014	60/60	50.7 ± 11.6	Acute	77/43	MSC	Routine	280.75	Intravenous	12	NIHSS, Barthel
Zhang et al. [22]	2015	14/28	57.2 ± 7.3	X	34/26	NSC	Routine	40	Intravenous	6	FIM
		18/28	56.9 ± 7.5	X	34/26	NSC	Routine	40	Subarachnoid	6	FIM
Shen [23]	2015	16/16	52 ± 10.4	Acute	X	UC-MSC	Routine	1000	Intra-carotid	3	FIM
Cai et al. [24]	2015	21/21	61.4 ± 6.7	Chronic	27/15	MSC	Routine	150–600	Intravenous	6	FIM, FMA, Barthel

MSC: mesenchymal stem cell; NSC: neural stem cell; MNC: bone marrow mononuclear stem cells; PBSC: peripheral blood stem cell; UC-MSC: umbilical cord blood mesenchymal stem cell; X: unknown.

**Table 2:** Study quality or risk of bias report.

Author	Year	Random allocation	Allocation concealment	Blinding of participants and personnel	Blinding of outcome assessment	Complete outcome data	Nonselective reporting	No other bias risk
Bang et al. [9]	2005	√	√	√	√	√	√	√
Sun et al. [10]	2008	√	Χ	Χ	Χ	Χ	Χ	Χ
Niu et al. [11]	2010	√	Χ	Χ	Χ	Χ	Χ	Χ
Bhasin et al. [12]	2011	Χ	Χ	Χ	√	√	√	√
Deng et al. [13]	2012	Χ	Χ	Χ	Χ	Χ	Χ	Χ
He [14]	2012	√	Χ	Χ	Χ	Χ	Χ	Χ
Bhasin et al. [15]	2012	Χ	Χ	Χ	√	√	√	√
Xiao [16]	2013	√	Χ	Χ	Χ	Χ	Χ	Χ
Bhasin et al. [17]	2013	Χ	Χ	Χ	√	√	√	√
Liu et al. [18]	2014	√	Χ	Χ	Χ	Χ	Χ	Χ
Xie et al. [19]	2014	√	Χ	Χ	Χ	Χ	Χ	Χ
Chen et al. [20]	2014	√	√	√	√	√	√	√
Prasad et al. [21]	2014	√	√	√	√	√	√	Χ
Zhang et al. [22]	2015	√	Χ	Χ	Χ	Χ	Χ	Χ
Shen [23]	2015	√	Χ	Χ	Χ	Χ	Χ	Χ
Cai et al. [24]	2015	√	√	Χ	Χ	Χ	Χ	Χ

**Table 3:** The correlations of NIHSS and clinical variables across studies by subgroup and heterogeneity analysis.

Subgroup	Mean difference (95% CI)	*I* ^2^ (%)
Patients' characteristics		
Acute stoke	0.93 (−0.65, 2.51)	57
Chronic stroke	2.28 (1.47, 3.09)	6
Cell type		
MSC	1.25 (0.35, 2.14)	38
Non-MSC	2.9 (1.75, 4.05)	—
Route of delivery		
Intravenous injection	1.0 (−0.18, 2.17)	41
Non-IV	2.38 (0.77, 3.99)	71
Follow-up period		
<6 months	1.41 (0.76, 2.06)	0
≥6 months	2.07 (−1.10, 5.24)	82

**Table 4:** The correlations of Barthel and clinical variables across studies by subgroup and heterogeneity analysis.

Subgroup	Mean difference (95% CI)	*I* ^2^ (%)
Patients' characteristics		
Acute stoke	12.61 (2.39, 22.84)	84
Chronic stroke	7.19 (4.18, 10.20)	0
Cell type		
MSC	8.37 (4.83, 11.91)	63
Non-MSC	—	—
Route of delivery		
Intravenous injection	7.58 (3.85, 11.30)	30
Non-IV	12.56 (−3.16, 28.27)	91
Follow-up period		
<6 months	5.2 (4.06, 6.34)	—
≥6 months	9.52 (4.92, 14.13)	58
